# Total intravenous anesthesia induced and maintained by a combination of remimazolam and remifentanil without a neuromuscular blocking agent: a prospective, observational pilot study

**DOI:** 10.1186/s12871-022-01779-2

**Published:** 2022-07-26

**Authors:** Insun Park, Mincheul Cho, Sun Woo Nam, Jung-Won Hwang, Sang-Hwan Do, Hyo-Seok Na

**Affiliations:** Department of Anesthesiology and Pain Medicine, Seoul National University Bundang Hospital, 82, Gumi 173, Bundang, Seongnam, Gyeonggi 13620 Republic of Korea

**Keywords:** General anesthesia, Hysteroscopy, Neuromuscular blockade, Remimazolam, Remifentanil

## Abstract

**Background:**

A novel short-acting benzodiazepine, Remimazolam, has recently been approved for general anesthesia and sedation. Hence, we investigated the feasibility and safety of remimazolam during the induction and maintenance of general anesthesia without using a neuromuscular blocking agent (NMBA) in patients undergoing hysteroscopic surgery.

**Methods:**

This prospective observational study included 38 patients undergoing hysteroscopic surgery. Remimazolam and remifentanil were the main anesthetic agents without an NMBA, and a supraglottic airway was inserted to protect the airway. The induction time, amount of each anesthetic agent used during anesthesia, intraoperative bispectral index (BIS) hemodynamic parameters, and recovery profiles were measured.

**Results:**

General anesthesia was successfully administered to 37 patients using remimazolam and remifentanil without NMBA. The induction doses of remimazolam and remifentanil were 0.4 mg/kg (interquartile range [IQR] 0.34–0.47 mg/kg) and 1.07 μg/kg (IQR, 0.90–1.29 μg/kg), respectively. Additionally, the maintenance doses of remimazolam and remifentanil were 1.14 mg/kg/h (IQR, 0.88–1.55 mg/kg/h) and 0.06 μg/kg/min (IQR, 0.04–0.08 μg/kg/min), respectively. Intraoperative BIS values had risen temporarily > 60 in eight patients (21.6%) despite administration of 2 mg/kg/h of remimazolam; thus, they were treated with supplementary midazolam. The median recovery time was 7 min (IQR, 5–8 min) after 40 min (IQR, 40.0–57.5 min) of total mean anesthesia time. There was no correlation between the infusion dose of remimazolam and recovery profiles, such as recovery time, final BIS of anesthesia, modified observer assessment of alertness/sedation (OAA/S) scale or post-anesthesia recovery (PAR) score when arriving at the PACU, and length of stay in the PACU (all *P* > 0.05).

**Conclusion:**

Remimazolam can be combined with remifentanil without an NMBA in female patients who undergo hysteroscopic surgery, during which a supraglottic airway is a feasible method to protect the airway.

**Trial registration:**

The study protocol was registered at ClinicalTrials.gov (NCT05025410) on 27/08/2021.

## Background

Remimazolam is a recently developed intravenous anesthetic agent with a more rapid onset of action and faster recovery than other benzodiazepines, including midazolam [1, 2]. Remimazolam is rapidly hydrolyzed into an inactive metabolite (CNS7054) by tissue esterases, which shows lower affinity at the benzodiazepine site of GABA_A_ receptor [3]. Owing to the short half-life of remimazolam and the pharmacological inactivity of the metabolite, rapid recovery can be achieved using remimazolam [2].

In addition to its short half-life, remimazolam has several other advantages. In the case of midazolam, the cumulative effect of its long-acting metabolite causes a slower recovery of neuropsychiatric function than does propofol [4, 5]. In contrast, remimazolam’s context-sensitive half-time (CSHT) remains < 10 min even after prolonged continuous infusion, contributing to a lower likelihood of delayed recovery from general anesthesia [2]. Moreover, previous studies have shown that remimazolam has minimal inhibitory effects on cardiovascular and respiratory systems [6, 7]. Furthermore, similar to other benzodiazepines, the sedative effect of remimazolam is easily antagonized by flumazenil.

Despite its various advantages, owing to its recent development, few studies have explored the efficacy and safety profile of remimazolam as a general anesthetic [8–10] and sedative agent for medical or surgical procedures [1, 11–13]. In addition, to the best of our knowledge, no studies have reported the appropriate use of remimazolam for general anesthesia without using a neuromuscular blocking agent (NMBA).

Therefore, we investigated the feasibility and safety of remimazolam during the induction and maintenance of general anesthesia without using NMBA in patients undergoing hysteroscopic surgery.

## Methods

The study protocol was approved by the Institutional Review Board of the Seoul National University Bundang Hospital (B-2109–706-301) and registered at ClinicalTrials.gov (NCT05025410, 01/11/2021). This study was performed at Seoul National University Bundang Hospital in South Korea between November 2021 and January 2022. After obtaining written informed consent, we recruited patients for the present study. This study was conducted in accordance with the principles of the Declaration of Helsinki. All methods followed the Strengthening the Reporting of Observational Studies in Epidemiology guidelines [14].

Patients aged 20–70 years who were scheduled for elective hysteroscopic surgery under general anesthesia were enrolled in this study. Exclusion criteria included an American Society of Anesthesiology (ASA) class III-V, body mass index > 35 kg/m^2^, galactose intolerance, Lapp lactase deficiency, or glucose galactose malabsorption, dextran 40 hypersensitivity, acute angle-closure glaucoma, obstructive sleep apnea, alcohol or drug dependency, or allergy to benzodiazepines and opioids.

### General anesthesia protocol

Patients were treated with 0.02 mg/kg of intravenous midazolam in the preoperative holding area. Noninvasive blood pressure, electrocardiography, pulse oximetry, and bispectral index (BIS) (Medtronic, Minneapolis, MN, USA) were measured on arrival at the operating room. In addition, the initial modified observer assessment of alertness/sedation (OAA/S) score (Table 1) was measured [15].

**Table 1:** Modified observer assessment of alertness/sedation (OAA/S) score

Score	Response
5	Responds readily to name spoken in normal tone
4	Lethargic response to name spoken in normal tone
3	Responds only after name is called loudly or repeatedly
2	Responds only after mild prodding or shaking
1	Does not respond to mild prodding or shaking
0	Does not respond to noxious stimulus

Anesthesia was induced using remimazolam (Byfavo Inj., Hana Pharm Co., Ltd., Seoul, Korea) and remifentanil (Ultiva Inj., GlaxoSmithKline Manufacturing S.p.A., Parma, Italy). Remimazolam was administered at a rate of 6 mg/kg/h and remifentanil was administered by target-controlled infusion at 4 ng/ml of effect-site concentration during the induction of anesthesia. A supraglottic airway (LMA supreme; Teleflex, Westmeath, Ireland) was inserted if the following four conditions were satisfied, (1) BIS value < 60, (2) modified OAA/S score = 0, (3) effect and plasma site concentration of remifentanil = 3 ng/ml; (4) loss of spontaneous breathing. If involuntary movements appeared during SGA insertion, we discontinued this process, rechecked the above four conditions, and then tried to insert the SGA again. If the same event occurred on the second attempt, 10 mg of rocuronium was administered intravenously, and the patient was excluded from the study.

The intraoperative target BIS was 40–60 to maintain an appropriate depth of anesthesia. According to the BIS value, remimazolam was administered at 1–2 mg/kg/h. If the BIS increased to ≥ 60 despite the maximal infusion rate of remimazolam, we administered 0.02 mg/kg of midazolam intravenously as a rescue dose, which was allowed twice intraoperatively. Nevertheless, if the BIS persisted ≥ 60, main anesthetic agent was changed from remimazolam to desflurane, and the case was excluded from the study.

Remifentanil was maintained within the range of 2–6 ng/ml of effect site concentration to maintain systolic arterial pressure within 20% of the baseline value. The patient was treated with the following medications when the systolic arterial pressure was outside the target range despite dose adjustment of remifentanil. For hypotension, 10–20 μg of phenylephrine was administered. If hypotension was accompanied by bradycardia < 50 beats/min, 5–10 mg of ephedrine was administered, and 0.5–1 mg of nicardipine was administered for hypertension. If hypertension with tachycardia > 100 beats/min, 2.5–5 mg of labetalol was administered. Tachycardia was treated with 5–10 mg of esmolol and bradycardia with 0.5 mg of atropine.

If patient movement occurred during surgical stimulation despite administration of both remimazolam and remifentanil at the set maximal dose, 10 mg of rocuronium was administered, and the patient excluded from the study.

### Recovery protocol

At the end of the surgery, remimazolam and remifentanil were discontinued. When remimazolam infusion stopped, the BIS value was recorded as the final BIS of anesthesia. Recovery was defined as satisfaction of the following four conditions, and then the SGA was removed: (1) BIS > 80; (2) modified OAA/S scale > 3; (3) remifentanil Ce < 1 ng/ml; (4) spontaneous breathing. The total dose of remimazolam and remifentanil was measured. If recovery was delayed 15 min, even after discontinuation of remimazolam, 0.2 mg of flumazenil was administered.

If an NMBA was administered during the operation, it was reversed with 1.0 mg of glycopyrrolate and 1.5 mg of neostigmine, or sugammadex (200 mg) according to the neuromuscular block status.

### Postanesthesia care unit (PACU) care

Modified OAA/S and post-anesthesia recovery (PAR) scores were measured as soon as patients arrived at the PACU and every 10 min thereafter. Particularly, the modified OAA/S score was evaluated at any time when the patient had a tendency to fall asleep. If the modified OAA/S score was < 2 in the PACU, flumazenil was administered. In addition, the incidence of immediate postoperative nausea and vomiting (PONV) within 1 h after surgery was examined.

### Outcome variables

This study focused on the feasibility of total intravenous anesthesia using remimazolam and remifentanil without NMBA. Therefore, various anesthesia induction-, maintenance-, and recovery-related parameters were evaluated as outcome variables. The time to a modified OAA/S score of 0 and time to BIS < 60 were recorded. The dose and infusion rate of remimazolam and remifentanil administered until the SGA was properly inserted and during general anesthesia were measured. In addition, intraoperative BIS values and recovery profiles were evaluated, such as recovery time, modified OAA/S and PAR score measured when arriving at PACU. Recovery time was defined as the interval from the cessation of remimazolam administration to the extubation of the SGA.

### Statistical analysis

Considering the minimum sample size to assume a normal distribution, 30 patients were initially targeted, and finally, 38 patients were recruited, assuming a dropout rate of 20%. The normal distribution of continuous variables was evaluated using the Shapiro–Wilk test. Normally distributed continuous variables are presented as mean (standard deviation) and if the distribution was not normal, median (interquartile range, IQR) was presented. Correlations between remimazolam infusion dosage and intraoperative hemodynamic and postoperative parameters were evaluated using Spearman’s correlation coefficient ($\uprho$). All statistical analyses were performed using SPSS software; version 25 (IBM, Chicago, IL, USA). Values were considered statistically significant at *P* < 0.05.

## Results

A total of 38 patients were enrolled in the study, and one patient dropped out. One drop-outed patient required rocuronium due to intraoperative movement, and the anesthetic agent was converted from remimazolam to desflurane because of increased BIS. The characteristics of 37 patients, surgery, and anesthesia are summarized in Table 2.

**Table 2:** The characteristic of patients, surgery, and anesthesia

Age (years)	48.7 ± 10.1
Height (cm)	159.2 ± 4.8
Weight (kg)	58.7 ± 10.5
BMI (kg/m^2^)	22.6 (20.7–24.4)
ASA I/II (%)	20/17 (54.1/45.9)
Diagnosis/Operation name	
Polyp of endometrium/Endometrial polypectomy	32 (86.5)
Myoma uteri/Hysteroscopic removal of leiomyoma	4 (10.8)
Vaginal bleeding/Diagnostic hysteroscopic operation	1 (2.7)
Time to modified OAA/S scale 0 (s)	63.0 (54.0—76.8)
Time to BIS < 60 (s)	135.0 (114.0—178.0)
Remimazolam dose until SGA insertion (mg/kg)	0.40 (0.34—0.47)
Remifentanil dose until SGA insertion (μg/kg)	1.07 (0.90 – 1.29)
Infusion rate of remimazolam during anesthesia maintenance (mg/kg/h)	1.14 (0.88 – 1.55)
Infusion rate of remifentanil during anesthesia maintenance (μg/kg/min)	0.06 (0.04 – 0.08)
Total anesthesia time (min)	40.0 (40.0–57.5)
Recovery time (min)	7 (5–8)
PACU length of stay (min)	30.0 (22.5–34.5)

*ASA* American society of anesthesiologists physical status, *BMI* Body mass index *OAA/S* Observer's assessment of alertness/sedation, *BIS* Bispectral index, *PACU* Postanesthesia care unit, *SGA* Supraglottic airway

Data are expressed as mean ± SD, median (IQR), or number (%)

**Fig. 1:**
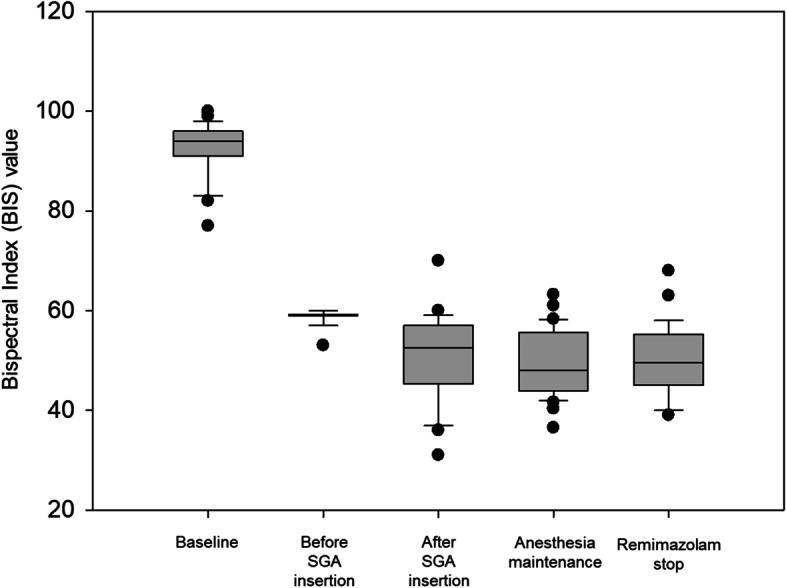
Intraoperative changes of bispectral index BIS, bispectral index; SGA, supraglottic airway

Intraoperative BIS is presented in Fig. 1. Immediately after administration of 0.02 mg/kg of intravenous midazolam, the median BIS value was 94. Total intravenous anesthesia using remimazolam and remifentanil led to intraoperative median BIS < 60; however, eight (21.6%) patients required supplementary midazolam during the anesthesia maintenance period because the BIS had risen to > 60 despite the maximum dose of remimazolam. During insertion an SGA without an NMBA, none of the patients exhibited involuntary movements or airway reflexes. Approximately 0.4 mg/kg remimazolam and 1.07 μg/kg remifentanil was administered until successful SGA insertion was ensured. At discontinuation of all remimazolam and remifentanil infusions, the final median BIS value was 50.0 (IQR, 45.0–55.5), and recovery time was 7 (IQR, 5–8) min.

During total intravenous anesthesia (TIVA) with remimazolam and remifentanil, the median systolic, diastolic, and mean arterial pressures were 102.0 (IQR, 97.2–108.8), 64.9 (IQR, 59.3–69.3), and 76.1 (IQR, 72.1–83.9) mmHg, respectively. The median heart rate was 66.3 (IQR, 60.5–71.1) beats/min. In all, four (10.8%) patients experienced intraoperative hypotension without bradycardia, and four (10.8%) patients presented with hypotension and bradycardia simultaneously. According to the pattern of hypotension and bradycardia, there was no significant difference when comparing the cumulative doses of remimazolam and remifentanil in the three subgroups (Table 3). Phenylephrine and ephedrine were administered to 5 and 4 patients, and their mean doses were 40.0 (25.3) μg and 5.0 (0.0) mg, respectively. None of the patients had received atropine or any medication for hypertension or tachycardia.

**Table 3:** The cumulative dose of remimazolam and remifentanil according to the presentation of intraoperative hypotension or bradycardia

Cumulative dose	Patients without hypotension and bradycardia (*n* = 29)	Patient with hypotension and without bradycardia (*n* = 4)	Patient with hypotension and bradycardia (*n* = 4)	*P*-value
Remimazolam (mg)	72.0 (60.0–89.5)	67.5 (45.5–127.0)	77.5 (53.5–111.3)	0.257
Remifentanil (μg)	213.0 (173.5–278.0)	224.5 (182.0–296.3)	157.0 (117.0–233.0)	0.736

Data are expressed as median (IQR)

During recuperation of patients from TIVA with remimazolam and remifentanil, two (5.4%) patients received flumazenil because it required > 15 min to meet the recovery criteria. On arrival at the PACU, the median modified OAA/S and PAR scores were 4 (4–5) and 8 (7–9). However, one (2.7%) patient who had not received flumazenil in the operating room required flumazenil in the PACU because the modified OAA/S scale decreased to 1. Patients who received flumazenil recovered within minutes.

No significant correlation was found between the intraoperative infused dose of remimazolam and postoperative recovery profiles, such as recovery time, final BIS of anesthesia, modified OAA/S scale or PAR score when arriving at the PACU, and length of stay in the PACU (all *P* > 0.05) (Table 4).

**Table 4:** Correlation between the total infused dose of remimazolam and postoperative recovery profiles

Recovery profiles	Spearman correlation coefficient (${\varvec{\uprho}}$)	*P-*value
Recovery time	-0.035	0.837
Final BIS of anesthesia	-0.066	0.697
Modified OAA/S when arriving at PACU	0.007	0.965
PAR score when arriving at PACU	0.031	0.855
PACU length of stay	-0.039	0.817

*BIS* Bispectral index, *OAA/S* Observer’s assessment of alertness/sedation, *PAR* Postanesthesia recovery score, *PACU* Post-anesthesia care unit

No patient experienced injection pain or immediate postoperative nausea or vomiting in the PACU.

## Discussion

In this study, we prospectively evaluated the feasibility of TIVA using remimazolam and remifentanil without an NMBA, which has not been fully explored because of limited experience with remimazolam. General anesthesia was successfully induced and maintained, except in one patient who was obese with a BMI of 33.9. In this case, intraoperative movement occurred, and the intraoperative BIS increased to > 60 under the maximal dose of remimazolam and injection of rescue midazolam. Thus, the patient received NMBA with an anesthetic agent substituted with desflurane. Although there was only one dropout case with a high BMI in this study, further research should be conducted on the efficacy and safety of remimazolam anesthesia in obese patients.

During TIVA with remimazolam and remifentanil without NMBA, the maintenance dose of remimazolam was approximately 1.14 mg/kg/h, which did not exceed the recommended dose. However, eight patients were treated with supplementary midazolam when the BIS increased to > 60. Fortunately, intraoperative awareness did not occur in any patient. Notably, BIS monitoring has not been validated for monitoring the depth of anesthesia with remimazolam. The narcotrend index is also less suitable for monitoring sedation depth with remimazolam, whereas the electroencephalogram β-ratio seems to be suitable for monitoring anesthetic depth by remimazolam [16]. In the present study, we observed the responsiveness scores of both the modified OAA/S and BIS during the induction period. During administration of remimazolam at a rate of 6 mg/kg/h for the induction of anesthesia, approximately 63 s was required to achieve a modified OAA/S scale of 0; however, approximately twice (135 s) as long was required as the BIS dropped to < 60, which is normally recommended for general anesthesia. This result is similar to those of previous studies [17]. Further studies are needed to determine whether BIS can adequately estimate the depth of remimazolam-induced anesthesia.

Remimazolam is known to cause less cardiovascular depression than propofol during general anesthesia [10, 18]. Our study mainly consisted of ASA class I or II patients, and 21.6% of patients experienced hypotension, similar to the previous reports [18]. Although remimazolam is less hypotensive than propofol, it should be noted that the incidence of hypotension is high in vulnerable patients [17, 19]. In our study, bradycardia was not observed alone, which occurred in four (10.8%) patients with hypotension. When remimazolam was used for general anesthesia induction or maintenance, the incidence of bradycardia was reported at 0–6.7% [17, 18]. Bradycardia was also observed at varying frequencies during the procedural sedation (1–11%) [20–22]. However, in early pharmacodynamics study, heart rate reportedly increased by 28 ± 15% during remimazolam infusion [16]. Intraoperative heart rate seems to be affected by the type and amount of opioids administered together; thus, the incidence of bradycardia requires additional research. Although the numbers were too small to draw precise conclusions from our subgroup comparison, the infusion dose of remimazolam and remifentanil did not seem to affect the occurrence of hypotension or bradycardia.

In terms of the postoperative recovery profile, the median recovery time from discontinuation of remimazolam to extubation was approximately 7 min without flumazenil, which almost coincides with the CSHT of remimazolam [1]. The relatively constant CSHT of remimazolam allows for no cumulative effect, even after a prolonged continuous infusion [2]. Although the anesthesia times of most patients were < 60 min in our study, no correlation was found between the intraoperative infusion dose of remimazolam and recovery parameters. However, two patients did not regain consciousness 15 min after the discontinuation of remimazolam infusion. Both patients woke up instantly after receiving flumazenil in the operating room and did not fall asleep. Another patient who recovered well from general anesthesia without flumazenil administration in the operating room became drowsy again in the PACU. She was awake after 0.2 mg of flumazenil was administered in the PACU. In these three patients, the amount of drug used did not exceed the usual dose used for the other patients in our study. Considering the nonsignificant correlation between remimazolam dose and recovery time, it is presumed that there may be other causes not yet revealed as the cause of delayed recovery. Flumazenil, a benzodiazepine antagonist, antagonizes the effects of remimazolam. Thus, routine flumazenil injection at the end of surgery may provide a fast and reliable recovery from remimazolam anesthesia. However, Yamamoto et al. recently reported a case in which one patient fell asleep again after remimazolam was reversed with flumazenil [23]. They noted that the effects of remimazolam reappeared when the blood concentration of flumazenil decreased.

As reported previously [1], vascular pain during remimazolam injection did not occur in our patients. In addition, except for three patients, all patients recovered from anesthesia without the use of an antagonist and there was no incidence of immediate PONV in the PACU.

The strength of this study is that it was the first to evaluate whether TIVA induced and maintained by a combination of remimazolam and remifentanil can be safely performed in surgery without use of a neuromuscular block. Tracheal intubation and several surgeries were performed under general anesthesia without neuromuscular blockade and the proper doses of various anesthetic agents were evaluated [24–27]. It was confirmed that remimazolam could be safely used as the main anesthetic under these conditions.

This study had several limitations. First, this study was a prospective, open-label, single-arm study. Thus, to validate our findings, non-inferiority or superiority studies between remimazolam and other anesthetic agents, such as propofol or volatile anesthetic gas, are warranted. Second, this study was conducted at a single tertiary university hospital and all patients were women who underwent hysteroscopy. Therefore, the generalizability of our findings is unclear. Hence, it is necessary to perform a study on male, old, or obese patients. Last, as the sample size of this single-arm study was not estimated, caution is required when interpreting the results.

## Conclusions

Remimazolam could be combined with remifentanil without NMBA in female patients who undergo hysteroscopic surgery, during which a SGA is a feasible method of protecting the airway. Future studies are required in various patients to compare remimazolam with other anesthetic agents, such as propofol and volatile anesthetic gases.

## Data Availability

The datasets generated and/or analyzed during the current study are not publicly available due to the restriction of IRB, but are available from the corresponding author on reasonable request.

## Abbreviations

ASA: American society of anesthesiology
BIS: Bispectral index
CSHT: Context-sensitive half-time
IQR: Interquartile range
NMBA: Neuromuscular blocking agent
OAA/S: Observer assessment of alertness/sedation
PACU: Post-anesthesia care unit
PAR: Post-anesthesia recovery
PONV: Postoperative nausea and vomiting
SGA: Supraglottic airway
TIVA: Total intravenous anesthesia
